# Serum CHI3L1 levels correlate with disease activity in rheumatoid arthritis and reveal potential molecular mechanisms

**DOI:** 10.3389/fimmu.2025.1729989

**Published:** 2025-12-18

**Authors:** Yi Deng, Dan Wang, Chao Wang, Kaiyun Guo, Ming Lei, Yanzhao Huang, Langui Tang, Ya Ding, Yan Gao

**Affiliations:** 1 Changde Hospital, Xiangya School of Medicion, Central South University (The First People’s Hospital of Changde City), Changde, China; 2 Hengyang Medical College, University of South China, Hengyang, China

**Keywords:** CHI3L1, immune infiltration, molecular docking, rheumatoid arthritis, single-cell RNA sequencing, WGCNA

## Abstract

**Objective:**

This study aims to elucidate the molecular regulatory mechanisms of Chitinase-3-like protein 1 (CHI3L1) in rheumatoid arthritis (RA) and its association with disease activity, focusing on its translational potential in RA diagnosis, dynamic monitoring, and precision therapy.

**Methods:**

Transcriptomic datasets (GSE77298, GSE89408) and single-cell RNA-seq data (GSE200815) were obtained from Gene Expression Omnibus (GEO). CHI3L1 expression was analyzed by Wilcoxon test, and diagnostic accuracy by Receiver Operating Characteristic (ROC) curve. Single-cell analysis defined cell type–specific expression of CHI3L1. Differential analysis combined with weighted gene co-expression network analysis (WGCNA) identified CHI3L1-related genes, followed by protein-protein interaction (PPI) and enrichment analyses. Immune infiltration was estimated with CIBERSORT, and competing endogenous RNA (ceRNA)/transcription factor networks were constructed to explore upstream regulation. Drug databases and molecular docking were integrated to predict therapeutic candidates. Clinically, serum CHI3L1 was measured by chemiluminescence immunoassay (CLIA) in RA patients (n=102) and controls (n=79), stratified by 28-joint Disease Activity Score with erythrocyte sedimentation rate (DAS28-ESR), and correlated with C-reactive protein (CRP), rheumatoid factor (RF), anti-cyclic citrullinated peptide antibody (CCP), and ESR.

**Results:**

CHI3L1 expression was significantly higher in RA across datasets (P<0.01) with strong diagnostic performance (AUC>0.8). Single-cell analysis revealed predominant fibroblast expression. Integrated analysis identified 51 candidate genes, enriched in chemokine signaling and mineral absorption pathways. PPI analysis highlighted TIMP1 and AQP9 as key genes, both strongly correlated with CHI3L1 (r>0, P<0.001). Immune infiltration showed increased M0 macrophages and plasma cells, reduced regulatory T cells, and significant correlations with CHI3L1. The ceRNA network indicated involvement of multiple miRNAs and lncRNAs. Drug prediction identified glibenclamide with the lowest binding energy (-9.386 kcal/mol). Clinically, serum CHI3L1 was markedly elevated in RA (P<0.001) with excellent diagnostic accuracy (AUC = 0.907). Higher CHI3L1 levels were observed in high-activity patients (P<0.01). CHI3L1 correlated with CRP (r=0.40, P<0.001), ESR (r=0.35, P<0.001), and moderately with CCP (r=0.21, P<0.05).

**Conclusion:**

This exploratory study suggests that CHI3L1 is a fibroblast-enriched molecule closely associated with immune dysregulation and RA activity, showing promise as a diagnostic and monitoring biomarker and a potential therapeutic target, though further validation through functional experiments and prospective studies is warranted.

## 1 Introduction

RA is a chronic systemic autoimmune disease characterized by persistent synovitis and progressive joint destruction, leading to irreversible functional impairment, reduced quality of life, and a substantial public health and economic burden (1, 2). Globally, RA affects approximately 0.5~1% of the adult population, with notable variation in prevalence across ethnic and geographic groups (3). Clinically, RA typically presents as symmetrical polyarthritis accompanied by pain, swelling, and morning stiffness; without timely and effective intervention, progressive structural joint damage may develop (4). Although biologic and targeted therapies have markedly improved disease management, approximately one-third of patients still fail to achieve adequate disease control, underscoring the need for reliable biomarkers of disease activity and additional therapeutic targets (5).

CHI3L1, a glycoprotein markedly upregulated under inflammatory conditions, has been implicated in the pathogenesis of multiple immune-mediated disorders (6–8). Through interactions with receptors such as IL-13Rα2, RAGE, and syndecan-1/αVβ3 integrin, CHI3L1 regulates macrophage activation, proinflammatory cytokine release, apoptosis, and tissue remodeling (9). In RA, CHI3L1 is consistently overexpressed in synovial tissue and synovial fluid and is associated with elevated inflammatory cytokines and extracellular matrix degradation (10, 11). Circulating CHI3L1 levels have also been positively correlated with disease activity and radiographic joint damage, highlighting its potential as a diagnostic and prognostic biomarker (12, 13). However, the precise mechanistic role of CHI3L1 in RA pathogenesis and its relationship with disease activity remain insufficiently defined.

To address this knowledge gap, the present study aims to elucidate the key molecular regulatory networks of CHI3L1 in RA and evaluate its potential clinical utility as a biomarker for diagnosis and disease activity assessment. By integrating genomic, immunological, and clinical datasets, this study seeks to provide mechanistic evidence for the role of CHI3L1 in RA and explore its translational value in early diagnosis, disease monitoring, and precision therapeutic strategies.

## 2 Methods

### 2.1 Clinical information

A total of 102 patients (30 males and 72 females, aged 35–86 years) who were diagnosed with RA and treated at the Department of Rheumatology and Immunology, Changde First People’s Hospital, between May 2025 and August 2025, were enrolled in this study, and their clinical data are presented in **Supplementary Table S1**. The diagnosis of RA was based on the 2010 American College of Rheumatology/European League Against Rheumatism (ACR/EULAR) classification criteria (14). In addition, 79 age- and sex-matched healthy individuals were recruited as controls. Exclusion criteria included the presence of other autoimmune diseases, active infections, malignancies, or severe hepatic and renal dysfunction. For all participants, fasting peripheral blood samples were collected in the morning, and serum was separated and stored in the institutional bio-bank. Serum CHI3L1 levels were measured using CLIA, and the assay kit used was the CHI3L1 Detection Kit (CLIA, REF: C86111) manufactured by Shenzhen YAHUILONG Biotechnology Co., Ltd. This kit has a linear range of 1.5–2000 ng/ml. The assay process was carried out in accordance with the manufacturer’s instructions. Disease activity in RA patients was assessed using the 28-joint Disease Activity Score with erythrocyte sedimentation rate (DAS28-ESR) (15), and patients were classified into three groups accordingly: (1) remission, DAS28-ESR < 2.6; (2) low to moderate activity, 2.6 ≤ DAS28-ESR ≤ 5.1; and (3) high activity, DAS28-ESR > 5.1.

### 2.2 Data acquisition and preprocessing

The RA-related transcriptomic datasets GSE77298 and GSE89408 were downloaded from the Gene Expression Omnibus (GEO) database (https://www.ncbi.nlm.nih.gov/geoprofiles/). GSE77298, comprising 16 RA synovial tissue samples and 7 normal controls, was used as the training set, whereas GSE89408, consisting of 150 RA and 23 normal synovial tissue samples, served as the validation set. For these microarray datasets, preprocessing steps included: (1) Normalization: log2 transformation was performed on the raw expression matrices to ensure data conformed to normal distribution assumptions; (2) Gene annotation conversion: probe IDs were converted to gene symbols, and when multiple probes mapped to the same gene, the probe with the highest median expression level was selected; (3) Background correction and batch effect removal. In addition, the single-cell RNA sequencing dataset GSE200815 was obtained from the GEO database. This dataset, generated on the GPL24676 platform, contained four RA synovial samples. Preprocessing of single-cell data was performed using 10x Genomics Cell Ranger for comparison, quantification, and cell identification.

### 2.3 Transcriptomic analysis of CHI3L1 expression and diagnostic efficacy in RA

Based on the transcriptomic datasets GSE77298 and GSE89408, the Wilcoxon rank-sum test was applied to perform nonparametric analysis of CHI3L1 expression between the RA and normal control (NC) groups. A P value < 0.05 was considered statistically significant. In addition, ROC curve analysis was used to evaluate the diagnostic performance of CHI3L1 expression in RA. The ROC curve was constructed by plotting sensitivity against specificity, with a larger area under the curve (AUC) indicating greater diagnostic accuracy.

### 2.4 Single-cell transcriptomic analysis

Single-cell transcriptomic data were analyzed using Seurat (v4.0.1) following the standard Seurat workflow (16). First, quality control was performed to filter high-quality cells according to the following criteria: nFeature RNA > 200, nCount RNA < 20,000, and percent.mt < 5%, to exclude low-quality cells and potential doublets. Gene expression levels were then normalized using the “NormalizeData” function with the “LogNormalize” method, which divided the expression count of each cell by its total UMI count and multiplied by a scale factor of 10,000. The top 2,000 highly variable genes, which exhibited the greatest expression variation across cells, were identified using the “FindVariableFeatures” function for subsequent clustering analyses. The expression matrix of these variable genes was scaled and centered using the “ScaleData” function through Z-score normalization, and principal component analysis (PCA) was performed for dimensionality reduction. Based on the Elbow plot and cumulative variance contribution rate, the top 20 principal components were selected for downstream analysis. Cell clustering was conducted with the “FindNeighbors” and “FindClusters” functions, combining the shared nearest neighbor (SNN) graph and the Louvain algorithm, with a resolution parameter set to 0.4 based on clustering rationality, followed by cell type annotation. Cell type annotation was performed based on the expression of canonical marker genes as previously described by Floudas et al. (17), by evaluating marker gene expression patterns across clusters and cross-referencing with established synovial tissue cell atlases. Finally, nonlinear dimensionality reduction and visualization were performed using the “RunUMAP” function, generating uniform manifold approximation and projection (UMAP) plots to display the distribution and relationships of cell subpopulations.

### 2.5 Identification of CHI3L1-associated candidate genes

To systematically identify genes closely associated with CHI3L1 expression and RA pathogenesis, an integrated analytical strategy combining differential expression analysis and WGCNA was employed (18, 19). First, differential expression analysis was performed on the training dataset using the limma package. Genes with an absolute fold change (|FC|) > 2.5 and a P value < 0.01 were considered differentially expressed genes (DEGs). Volcano plots and heatmaps were generated to visualize the DEGs. Subsequently, WGCNA was performed to identify RA-associated co-expression modules and key hub genes. The optimal soft-thresholding power (β) was first determined using the pickSoftThreshold function, after which an adjacency matrix was constructed and transformed into a topological overlap matrix (TOM) to quantify the similarity of gene co-expression. Hierarchical clustering was then conducted on the TOM-based dissimilarity matrix, and gene modules were identified using the dynamic tree cut algorithm, with parameters set as follows: minimum module size = 30, cut height = 3, and maximum module distance = 0.4. Next, Pearson correlation analysis was applied to assess the relationships between module eigengenes (MEs) and both disease status and CHI3L1 expression, and modules significantly associated with disease status and CHI3L1 expression were selected (P < 0.05). Hub genes were further screened based on two criteria: gene significance (GS) and module membership (MM), with thresholds of |GS| > 0.75 and |MM| > 0.75. Finally, DEGs were cross-referenced with WGCNA-derived hub genes to identify overlapping genes, which were considered candidate genes closely associated with CHI3L1 expression and RA progression for subsequent functional analysis and experimental validation. Additionally, the Benjamini-Hochberg method was employed to correct the false discovery rate (FDR) for the key differentially expressed genes among them.

### 2.6 Functional enrichment analysis of candidate genes

To elucidate the biological processes and signaling pathways associated with CHI3L1-related candidate genes, functional enrichment analyses were performed. Kyoto Encyclopedia of Genes and Genomes (KEGG) pathway and Gene Ontology (GO) enrichment analyses were conducted using the DAVID database (https://david.ncifcrf.gov) (20). GO annotations included biological processes (BPs), cellular components (CCs), and molecular functions (MFs). The degree of enrichment of key genes in each KEGG pathway and GO term was calculated, and Fisher’s exact test was applied to identify significantly enriched pathways and terms (P < 0.05) (21). Visualization of enrichment results was performed using Sangerbox (http://www.sangerbox.com/), a comprehensive online platform for bioinformatics analysis. In addition, based on expression profile data, gene set enrichment analysis (GSEA) was performed using the “gsea” R package to evaluate the differential expression of major signaling pathways between the RA and NC groups, thereby revealing RA-associated biological functions and pathways.

### 2.7 PPI network analysis and identification of key genes

The STRING database (https://cn.string-db.org/) was used to construct a PPI network of candidate genes (22). The resulting network was imported into Cytoscape software for visualization, and hub nodes were identified based on node degree (23). Genes directly interacting with CHI3L1 were extracted as primary targets of interest. Differential expression of these genes was then validated using both the GEO training and validation datasets, and genes exhibiting consistent expression trends across the two datasets were designated as key genes. Furthermore, ROC curve analysis was performed using the pROC package to calculate the AUC for evaluating the diagnostic performance of these key genes as biomarkers for RA (24). The expression correlations between CHI3L1 and the key genes were also calculated, and a correlation matrix was generated for visualization.

To enhance diagnostic accuracy and clinical applicability, a multi-gene diagnostic model was constructed based on the key genes identified through PPI and WGCNA analyses. The training dataset (GSE77298) and validation dataset (GSE89408) were first merged, and batch effects were removed using the ComBat algorithm from the sva package in R to ensure data compatibility. A nomogram prediction model was then established using the rms package, incorporating the expression levels of CHI3L1 and its related key genes as predictor variables. The nomogram quantified gene expression into a cumulative point system to calculate individual RA risk probabilities. Model performance was evaluated using ROC curve analysis with AUC calculation via the pROC package to assess discriminative ability. Additionally, decision curve analysis (DCA) was performed using the rmda package to evaluate the clinical utility and net benefit of the model across varying threshold probabilities, thereby determining its potential value in clinical decision-making for RA diagnosis.

### 2.8 Immune infiltration analysis

CIBERSORTx (https://cibersortx.stanford.edu/) is a machine learning algorithm based on gene expression profiles that can estimate the relative abundance of 22 human immune cell subsets within complex tissues. In this study, CIBERSORTx was applied to analyze the composition and relative proportions of immune cells in RA and control tissue samples (25). The Wilcoxon rank-sum test was then used to compare differences in immune cell infiltration between the RA and NC groups. Finally, Pearson correlation analysis was performed to assess correlations among immune cell subsets as well as between key genes and immune cell infiltration.

### 2.9 Construction of the ceRNA network and prediction of transcription factors

To further investigate the upstream regulatory mechanisms of the key genes, a ceRNA regulatory network was constructed. First, the miRWalk database (http://mirwalk.umm.uni-heidelberg.de/) was used to predict miRNAs interacting with the key genes (26). Next, the starBase database (http://starbase.sysu.edu.cn/) was applied to identify upstream lncRNAs targeting these miRNAs. Based on the predicted miRNA–mRNA and lncRNA–miRNA interactions, a ceRNA regulatory network was established and visualized using Cytoscape software, thereby providing an intuitive framework for elucidating the potential multilayered regulatory mechanisms of the key genes.

### 2.10 Drug prediction and molecular docking analysis

Key genes were uploaded to the Enrichr database (https://maayanlab.cloud/Enrichr/) for drug prediction using the DSigDB module, and further analyzed in the CMAP database (https://clue.io/) to identify potential therapeutic compounds (27). Predicted small-molecule drugs were then subjected to molecular docking analysis with key targets using AutoDock Vina to evaluate binding affinity and interaction patterns. The three-dimensional structures of candidate small molecules were downloaded from the PubChem database (https://pubchem.ncbi.nlm.nih.gov/). Based on the structural information of the target proteins, docking regions were defined and docking parameters were set to complete the simulations. For each key target, the ligand–receptor complex with the lowest binding energy was selected for visualization, providing an intuitive demonstration of the interactions between small molecules and target proteins, thereby supporting a deeper understanding of their potential mechanisms of action.

## 3 Results

### 3.1 Transcriptomic analysis of CHI3L1 expression and diagnostic efficacy in RA

**Figure 1:**
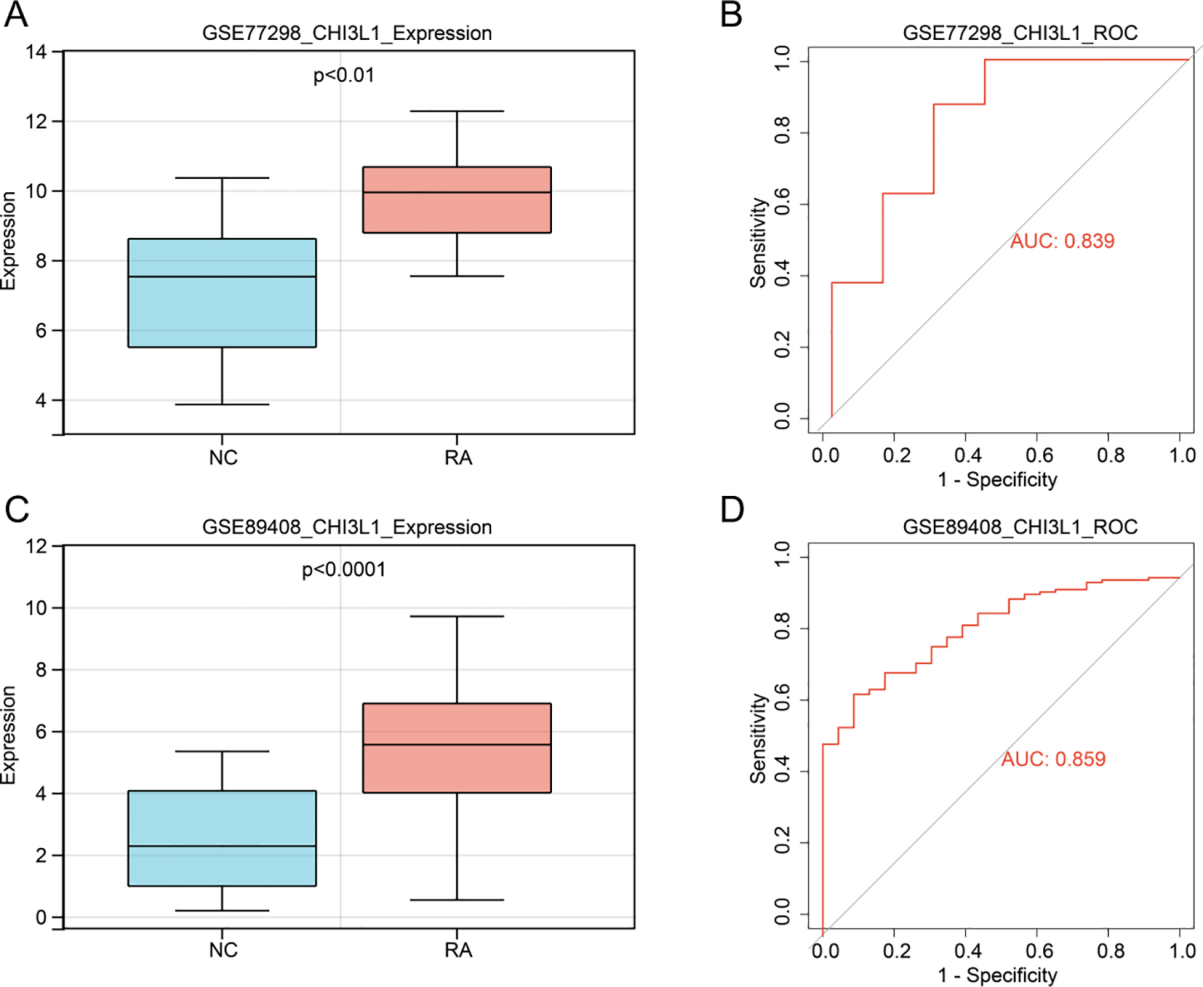
Transcriptomic analysis of CHI3L1 expression and diagnostic efficacy in RA. In the GSE77298 dataset, differential expression of CHI3L1 between RA and NC groups **(A)** and the corresponding ROC curve analysis **(B)**. In the GSE89408 dataset, differential expression of CHI3L1 between RA and NC groups **(C)** and the corresponding ROC curve analysis **(D)**.

Based on the transcriptomic datasets GSE77298 and GSE89408, the Wilcoxon rank-sum test was used to analyze CHI3L1 expression between the RA and NC groups, and ROC curve analysis was performed to evaluate its diagnostic efficacy. Expression analysis revealed that CHI3L1 was significantly upregulated in the RA group compared with the NC group in both GSE77298 and GSE89408 (P < 0.01) (**Figures 1A, C**). ROC analysis further demonstrated that the AUC values for CHI3L1 in distinguishing RA from NC exceeded 0.8 in both datasets (**Figures 1B, D**,). These findings suggest that CHI3L1 may play an important role in the pathogenesis of RA and holds potential diagnostic value.

### 3.2 Single-cell transcriptomics reveals cellular heterogeneity and CHI3L1 expression patterns in RA

**Figure 2:**
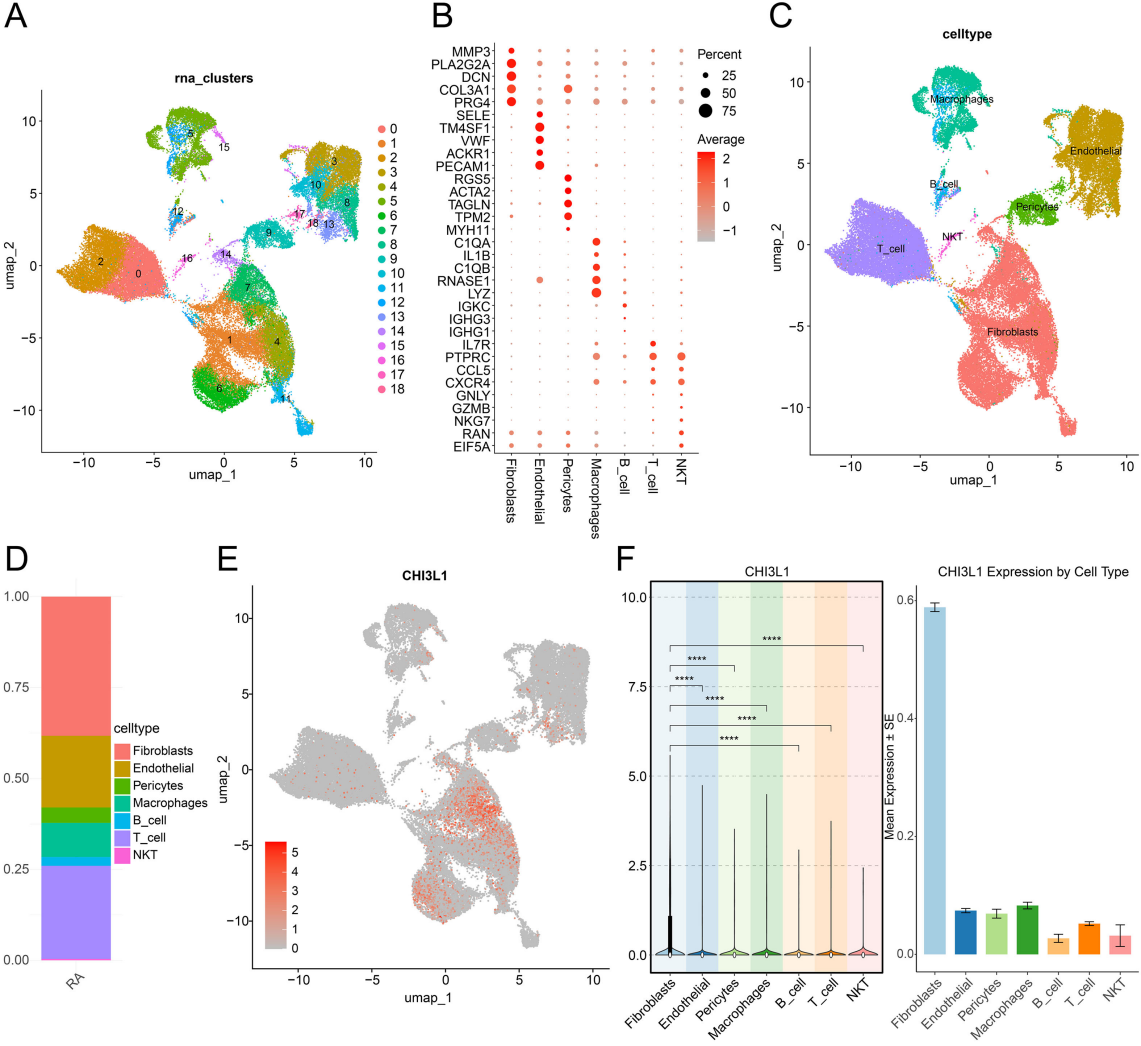
Single-cell transcriptomic analysis of RA synovial tissues. **(A)** UMAP visualization of distinct cell clusters identified from RA synovial samples. **(B)** Heatmap of canonical marker gene expression across different cell types; bubble size indicates the proportion of cells expressing the gene, and color intensity reflects average expression levels. **(C)** UMAP plot showing the annotation of major cell types based on known marker genes. **(D)** Proportional distribution of cell types in RA samples. **(E)** UMAP plot showing the expression pattern of CHI3L1 across all single cells; color intensity represents expression level. **(F)** Violin plot (left panel) and bar chart (right panel) illustrating CHI3L1 expression across different cell types; asterisks denote statistical significance (****P < 0.0001).

Quality control analysis was performed using the Seurat package to filter high-quality cells (nFeature_RNA > 200, nCount_RNA < 20,000, percent_mt < 5%) (**Supplementary Figures S1 A–C**). Across the four RA synovial samples (GSM6044092–GSM6044095), 53,242 cells were retained with total UMI counts of 4.40×10^7^, 2.18×10^7^, 5.35×10^7^, and 4.38×10^7^, and median UMI depths of approximately 2,500, 6,400, 2,000, and 1,400 per cell, respectively. These metrics indicated adequate sequencing depth and data quality for downstream analysis. The datasets were then integrated, normalized, and screened for highly variable genes. The top 2,000 most variable genes were selected for subsequent dimensionality reduction and clustering (**Supplementary Figure S1D**). PCA was used to capture major sources of variation, and the top 20 principal components were selected based on the elbow plot (**Supplementary Figure S1E**). Subsequently, UMAP was employed for nonlinear dimensionality reduction and clustering, which identified 19 cell subpopulations with distinct transcriptional profiles (**Figure 2A**). Cell type annotation was performed using canonical marker genes (**Figure 2B**), revealing seven major cell types, including fibroblasts, endothelial cells, pericytes, macrophages, B cells, T cells, and natural killer T (NKT) cells (**Figure 2C**). Analysis of cell composition demonstrated that fibroblasts were the most abundant population in RA samples, followed by endothelial cells and T cells (**Figure 2D**), suggesting that these cell types may be closely implicated in RA pathogenesis.

Further investigation of CHI3L1 expression revealed a clear enrichment in fibroblasts. UMAP visualization confirmed that CHI3L1 expression was predominantly localized to fibroblast clusters (**Figure 2E**), and both violin plot and bar chart analyses demonstrated that CHI3L1 expression levels in fibroblasts were significantly higher than in other cell types (**Figure 2F**), indicating a potential role in fibroblast activation during RA progression. These findings provide important cellular-level evidence for the pathogenic role of CHI3L1 in RA and suggest that fibroblast-derived CHI3L1 may be a key driver of disease progression.

### 3.3 Identification of CHI3L1-associated candidate genes in RA

**Figure 3:**
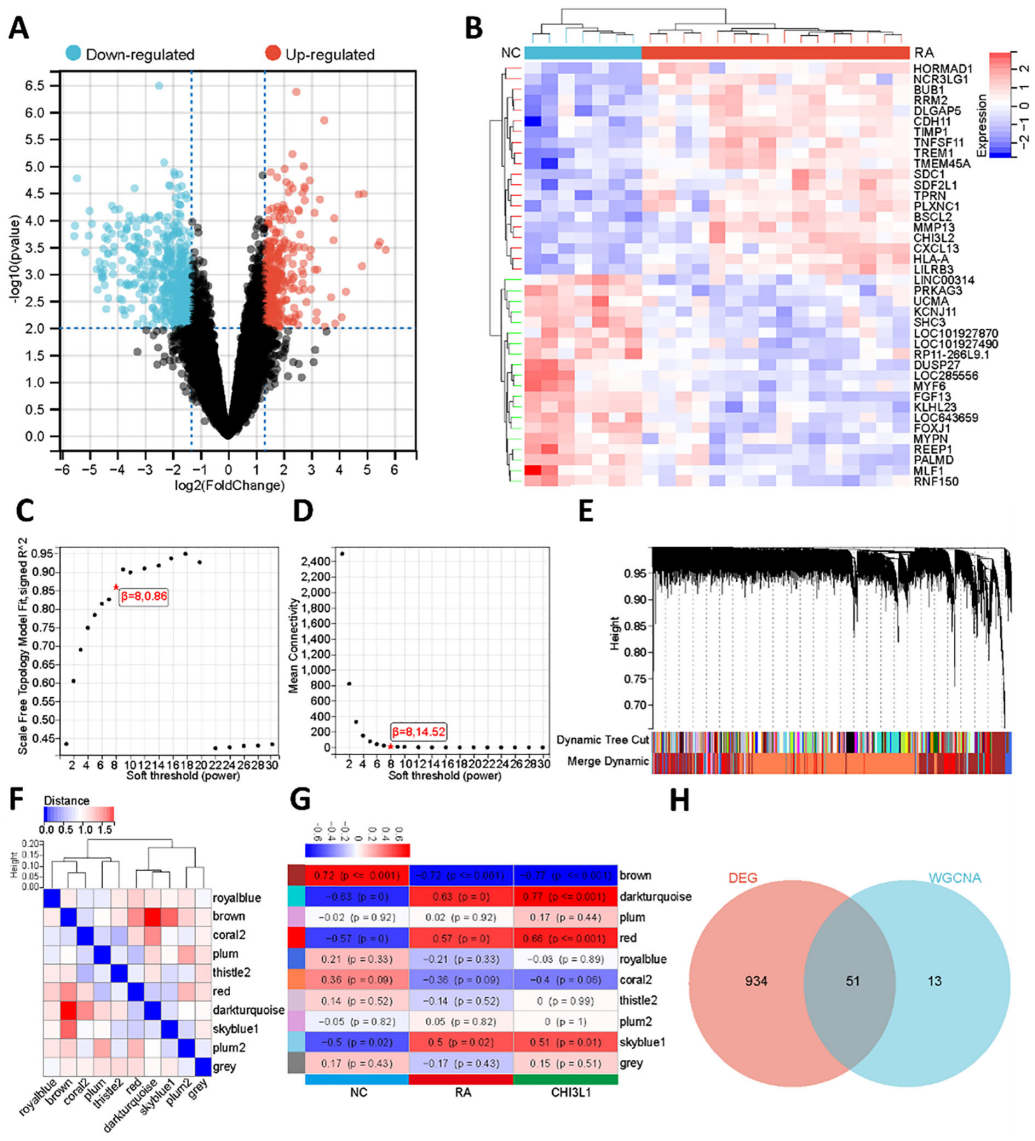
Transcriptome differences analysis between RA and NC groups and WGCNA. **(A)** Volcano plot showing DEGs between RA and NC groups. **(B)** Heatmap of the top 20 upregulated and downregulated genes. **(C)** The trend of the scale-free topological fitting index R^2^ varying with the soft threshold β. **(D)** The trend of the average connectivity varying with β. **(E)** Gene clustering tree and dynamic tree cut results, with different colors representing distinct co-expression modules. **(F)** Heatmap of correlations/distances among MEs, reflecting inter-module relationships. **(G)** Module–trait correlation heatmap showing the associations of MEs with disease status and CHI3L1 expression (numbers in squares represent correlation coefficients r, values in parentheses represent P values, and color intensity indicates correlation strength). **(H)** Venn diagram showing the intersection between WGCNA-derived hub genes and DEGs, identifying 51 key genes closely related to CHI3L1 in RA pathogenesis.

A total of 985 DEGs were identified in the RA groups compared with the normal groups, including 380 upregulated and 605 downregulated genes (**Figure 3A**). A heatmap illustrated the expression patterns of the top 20 upregulated and downregulated genes (**Figure 3B**). WGCNA was then performed. Using the pickSoftThreshold function, the optimal soft-thresholding power was determined as β = 8, after which an adjacency matrix was constructed and transformed into a TOM to represent gene co-expression similarity (**Figures 3C, D**). Hierarchical clustering combined with the dynamic tree cut algorithm (minimum module size = 30, deep split = 3, maximum module distance = 0.4) identified 10 distinct gene modules (**Figure 3E**). Analysis of module eigengene (ME) connectivity showed that inter-module distances were all greater than 0.4, indicating good independence among modules (**Figure 3F**). Correlation analysis of MEs with disease status and CHI3L1 expression revealed that the dark turquoise (disease status: r = 0.63, P < 0.001; CHI3L1: r = 0.77, P < 0.001), brown (disease status: r = -0.72, P < 0.001; CHI3L1: r = -0.77, P < 0.001), skyblue1 (disease status: r = 0.5, P < 0.05; CHI3L1: r = 0.51, P < 0.05), and red (disease status: r = 0.57, P < 0.001; CHI3L1: r = 0.66, P < 0.001) modules were significantly associated with disease state (**Figure 3G**). Under the thresholds of |MM| > 0.75 and |GS| > 0.75, a total of 64 hub genes co-expressed with CHI3L1 were identified. Intersection with DEGs yielded 51 overlapping genes (**Figure 3H**), which were considered key CHI3L1-related co-expressed genes in RA. After the FDR adjustment, CHI3L1 and the key differentially expressed genes still showed statistical significance, the relevant data can be found in the attached materials (**Supplementary Table S2**).

### 3.4 Functional enrichment analysis of overlapping genes

**Figure 4:**
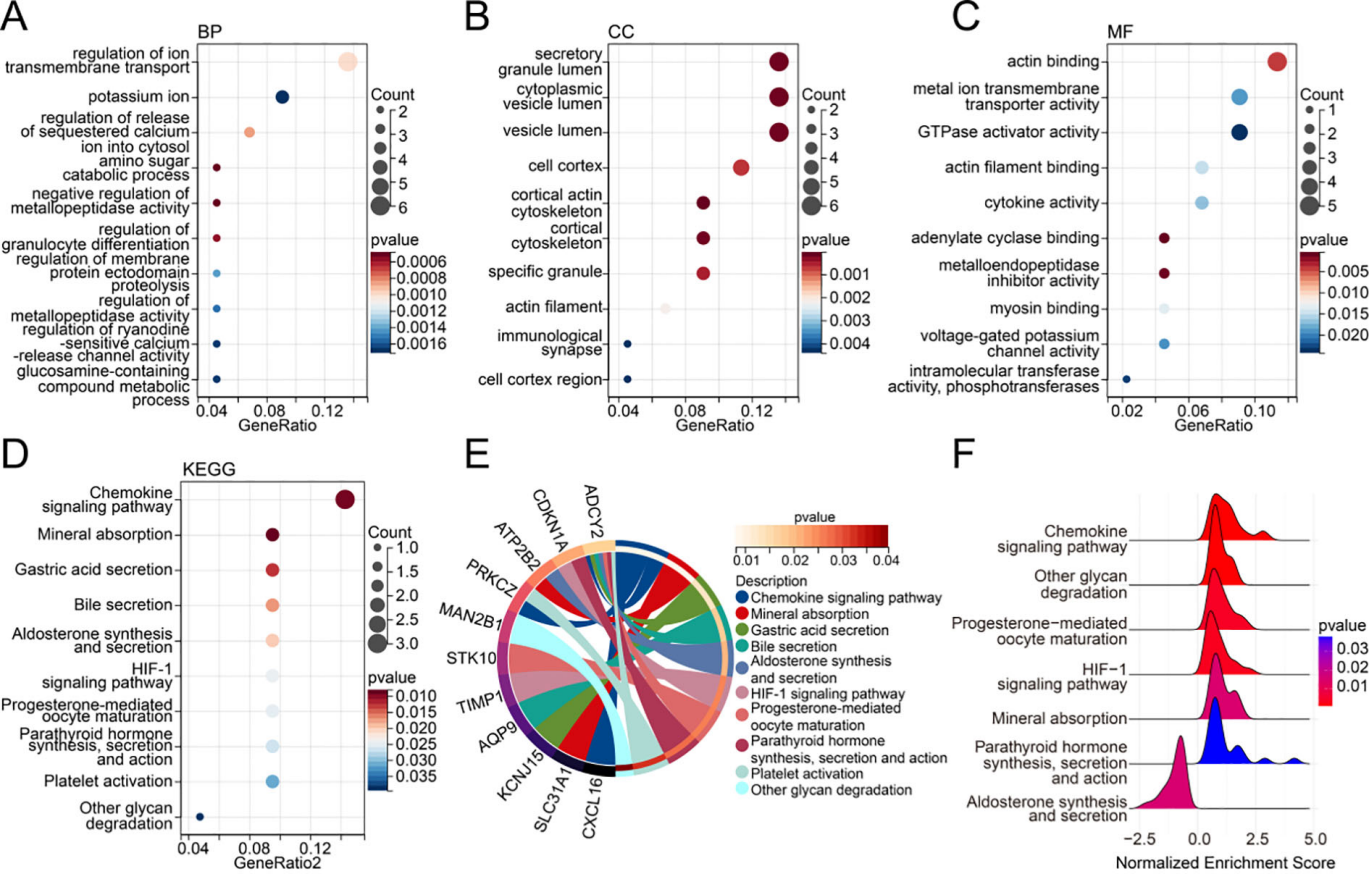
Functional enrichment analysis. **(A–C)** Bubble plots of GO enrichment for candidate genes, including biological processes (BP) **(A)**, cellular components (CC) **(B)**, and molecular functions (MF) **(C)**, showing the top 10 most significant terms. **(D)** Bubble plot of KEGG pathway enrichment for candidate genes. **(E)** Network diagram illustrating the relationships between co-expressed genes and enriched KEGG pathways. **(F)** Single-gene GSEA analysis of CHI3L1.

GO and KEGG enrichment analyses were performed on the 51 overlapping genes identified above. In the GO enrichment analysis, these genes were primarily enriched in biological processes (BPs) such as regulation of ion transmembrane transport, potassium ion transmembrane transport, and regulation of release of sequestered calcium ion into cytosol (**Figure 4A**); cellular components (CCs) such as secretory granule lumen, cytoplasmic vesicle lumen, and vesicle lumen (**Figure 4B**); and molecular functions (MFs) such as actin binding, metal ion transmembrane transporter activity, and GTPase activator activity (**Figure 4C**). KEGG pathway analysis revealed that these genes were involved in pathways including Chemokine signaling pathway, Mineral absorption, and Gastric acid secretion (**Figures 4D, E**).

In addition, single-gene GSEA analysis of CHI3L1 showed significant enrichment in multiple pathways, including Aldosterone synthesis and secretion, Chemokine signaling pathway, Other glycan degradation, Progesterone-mediated oocyte maturation, HIF-1 signaling pathway, Mineral absorption, and Parathyroid hormone synthesis, secretion and action (**Figure 4F**). Notably, except for the Aldosterone synthesis and secretion pathway, which was significantly enriched in the CHI3L1 low-expression group (NES < -1, P < 0.05), all other pathways were significantly enriched in the CHI3L1 high-expression group (NES > 1, P < 0.05). These findings suggest that CHI3L1 may exert its biological functions through the regulation of multiple signaling pathways, particularly those related to inflammation, glucose metabolism, hypoxic stress, and mineral metabolic homeostasis.

### 3.5 PPI network analysis of overlapping genes

**Figure 5:**
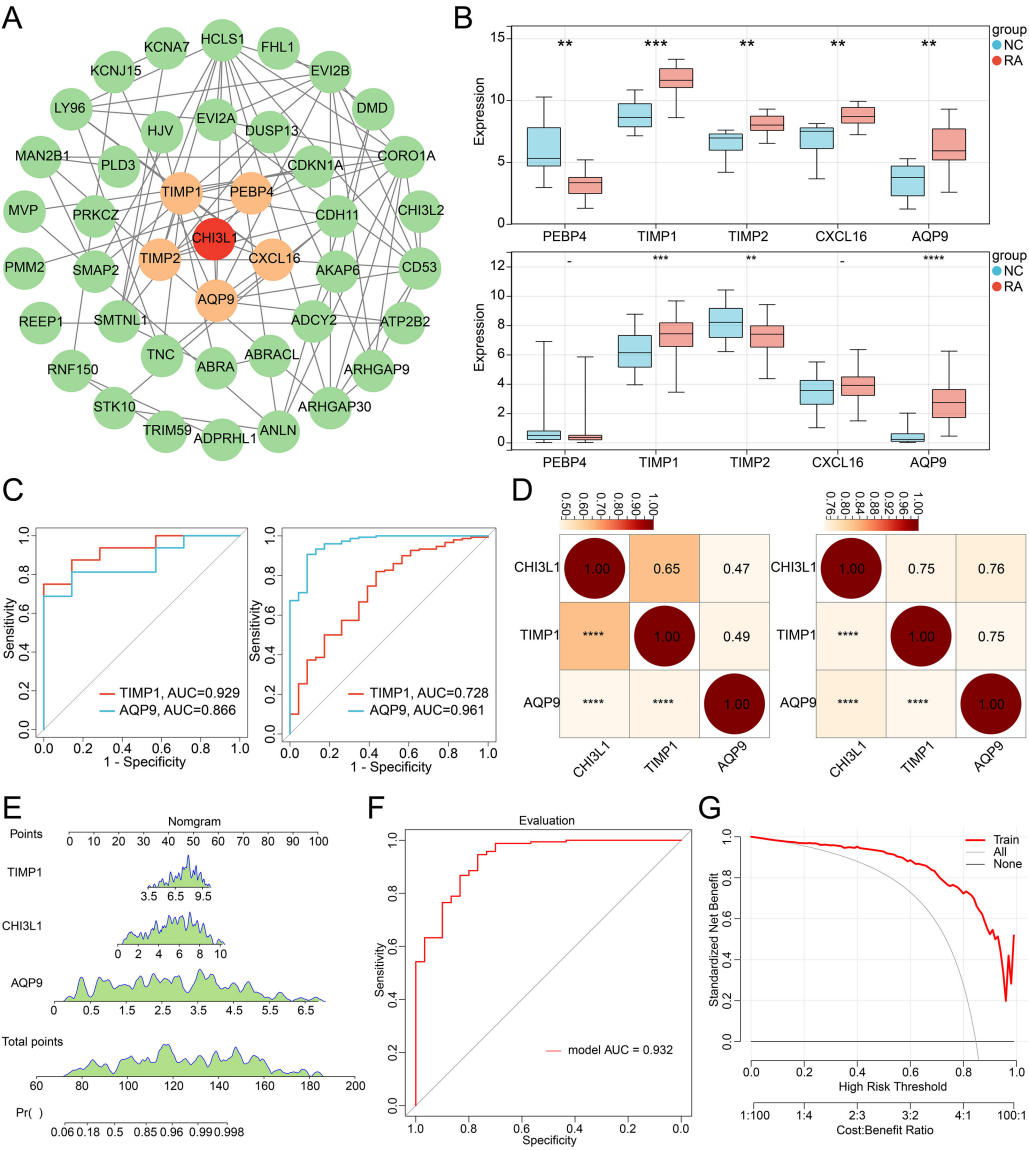
PPI network, differential expression, and correlation analysis of CHI3L1 and its directly associated genes. **(A)** PPI network of CHI3L1 co-expressed genes. Yellow circular nodes represent genes directly interacting with CHI3L1, and green circular nodes represent indirectly related genes; edges indicate protein–protein interactions. **(B)** Differential expression of CHI3L1-interacting genes in the training and validation datasets; asterisks indicate statistical significance (**P<0.01, ***P<0.001, ****P<0.0001). **(C)** ROC curves evaluating the diagnostic performance of key CHI3L1-interacting genes for RA, based on the training and validation datasets. **(D)** Correlation heatmaps of CHI3L1 with its directly associated key genes in both datasets; asterisks denote statistical significance (*P < 0.05; **P < 0.01; ***P < 0.001). **(E)** Nomogram prediction model integrating the expression levels of CHI3L1, TIMP1, and AQP9 for predicting individual RA risk probability. **(F)** ROC curve analysis evaluating the discriminative ability of the nomogram model. **(G)** DCA assessing the clinical net benefit of the nomogram model across different threshold probabilities.

The STRING database was used to analyze the interactions among the 51 overlapping genes and to construct a PPI network, aiming to explore their network-level regulatory roles in RA progression. The resulting PPI network comprised 42 nodes and 78 edges, representing 78 interaction relationships among 42 genes (**Figure 5A**). Notably, PEBP4, TIMP1, TIMP2, CXCL16, and AQP9 showed direct interactions with CHI3L1, suggesting that these genes may act as critical regulators in CHI3L1-mediated RA progression. Differential expression of CHI3L1-interacting candidate genes was then examined in both the training and validation datasets using the Wilcoxon rank-sum test. The results demonstrated that TIMP1 and AQP9 were significantly upregulated in RA samples compared with NC controls (P < 0.05) (**Figure 5B**), indicating that both genes may play important roles in RA pathogenesis. ROC curve analysis further revealed that TIMP1 and AQP9 effectively discriminated RA patients from normal controls, with AUC values exceeding 0.7 in both datasets (**Figure 5C**), highlighting their diagnostic potential. To further clarify the expression associations between CHI3L1 and these key regulatory genes, Pearson correlation coefficients were calculated. CHI3L1 expression was significantly positively correlated with both TIMP1 and AQP9 (P < 0.001) (**Figure 5D**), suggesting that CHI3L1 may participate in RA-related inflammatory and pathological processes through co-expression with these genes. Collectively, these findings provide important insights into the molecular mechanisms underlying CHI3L1-mediated RA progression and indicate that TIMP1 and AQP9 may serve as potential biomarkers for RA.

Given the strong associations identified through both PPI and WGCNA analyses, we further constructed a multi-gene diagnostic model to enhance the diagnostic accuracy for RA. After merging the training and validation datasets and removing batch effects, a nomogram prediction model was established based on CHI3L1, TIMP1, and AQP9 (**Figure 5E**), which quantified gene expression levels into a cumulative point system to calculate individual RA risk probabilities. ROC curve analysis demonstrated that the nomogram model achieved excellent discriminative ability with an AUC of 0.932 (**Figure 5F**), substantially outperforming individual biomarkers. Decision curve analysis further confirmed that the model provided high net benefit across a wide range of threshold probabilities (**Figure 5G**), indicating its substantial potential as a clinical decision-making support tool for RA diagnosis and enabling more informed treatment decisions based on individual risk assessment. Collectively, these findings not only provide important insights into the molecular mechanisms underlying CHI3L1-mediated RA progression but also establish a robust multi-gene diagnostic framework with superior diagnostic accuracy and clinical applicability for RA diagnosis and risk stratification.

### 3.6 Immunological analysis of CHI3L1 involvement in RA

**Figure 6:**
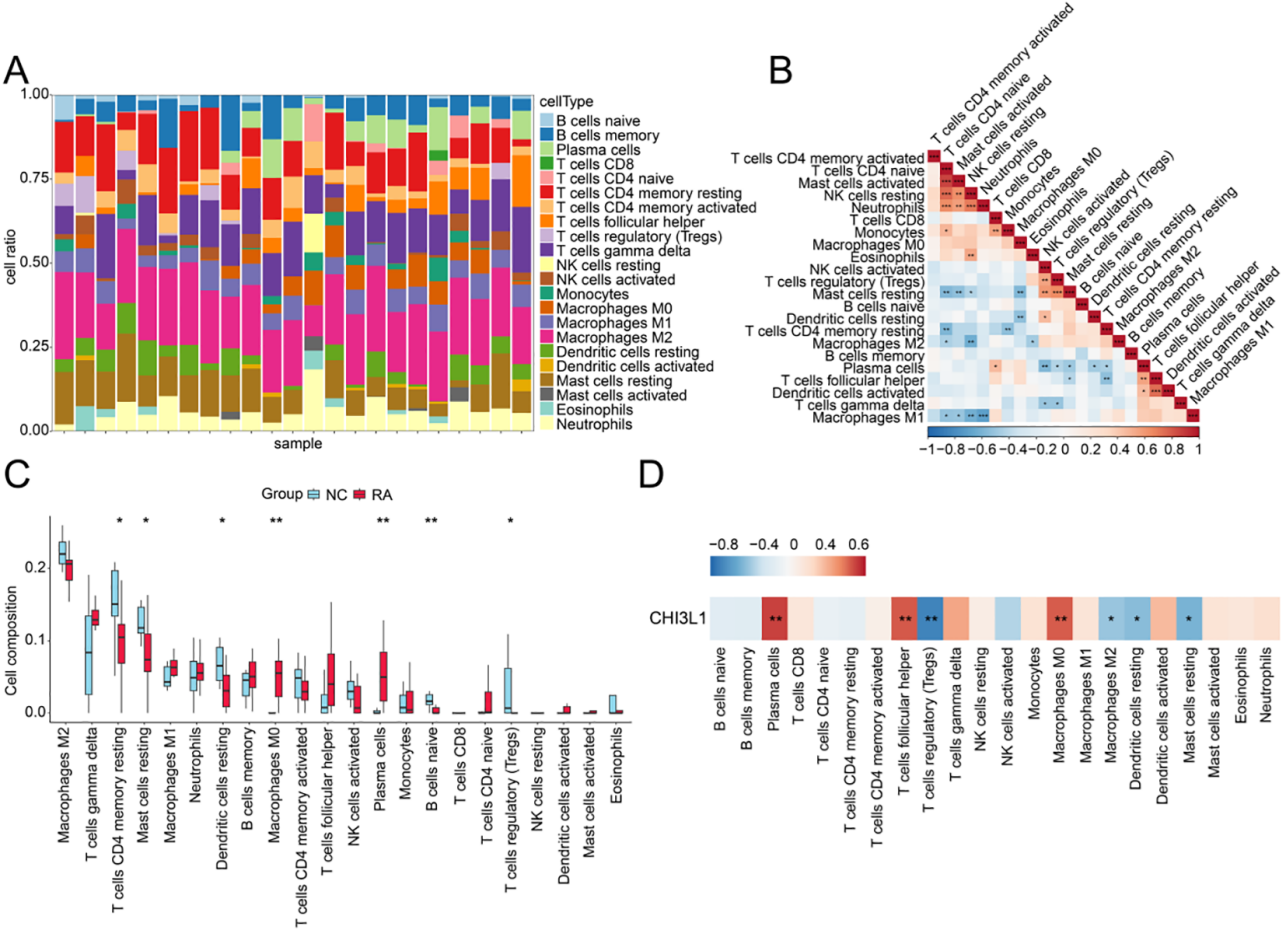
Immune infiltration analysis. **(A)** Proportions of immune cell infiltration across samples. **(B)** Correlation heatmap of differential immune cells. *P < 0.05, **P < 0.01, ***P < 0.001. **(C)** Differences in immune cell infiltration proportions between RA and NC samples. *P < 0.05, **P < 0.01, ***P < 0.001, ****P < 0.0001. **(D)** Correlation heatmap between key genes and immune cells. *P < 0.05, **P < 0.01.

Analysis of immune cell infiltration profiles revealed distinct differences in the types and proportions of infiltrating immune cells between NC and RA samples (**Figure 6A**). Correlation analysis revealed the strongest negative correlation between macrophages M1and neutrophils (r = –0.70, P < 0.001), while the strongest positive correlation was observed between T cells CD4 naïve and mast cells activated (r = 0.86, P < 0.001) (**Figure 6B**). Comparative analysis of immune cell subsets between NC and RA groups showed that macrophages M0 (P < 0.01) and plasma cells (P < 0.01) were upregulated in RA, whereas T cells CD4 memory resting (P < 0.05), mast cells resting (P < 0.05), dendritic cells resting (P < 0.01), B cells naïve (P < 0.01), and T cells regulatory (Tregs) (P < 0.05) were significantly downregulated (**Figure 6C**). Moreover, CHI3L1 expression was closely associated with the infiltration levels of multiple immune cell subsets, with significant correlations observed for seven subsets. Specifically, positive correlations were detected with plasma cells (r > 0, P < 0.01), T cells follicular helper (r > 0, P < 0.01), and macrophages M0 (r > 0, P < 0.01), while negative correlations were observed with T cells regulatory (Tregs) (r < 0, P < 0.01), macrophages M2 (r < 0, P < 0.05), dendritic cells resting (r < 0, P < 0.05), and mast cells resting (r < 0, P < 0.05) (**Figure 6D**). Taken together, these results suggest that the immunopathological mechanisms of RA may involve aberrant CHI3L1 expression, which reshapes the synovial immune microenvironment by regulating immune cell recruitment, activation, and migration, thereby inducing imbalances in specific immune cell subsets and promoting autoimmunity and chronic inflammation.

### 3.7 Construction of the regulatory network

**Figure 7:**
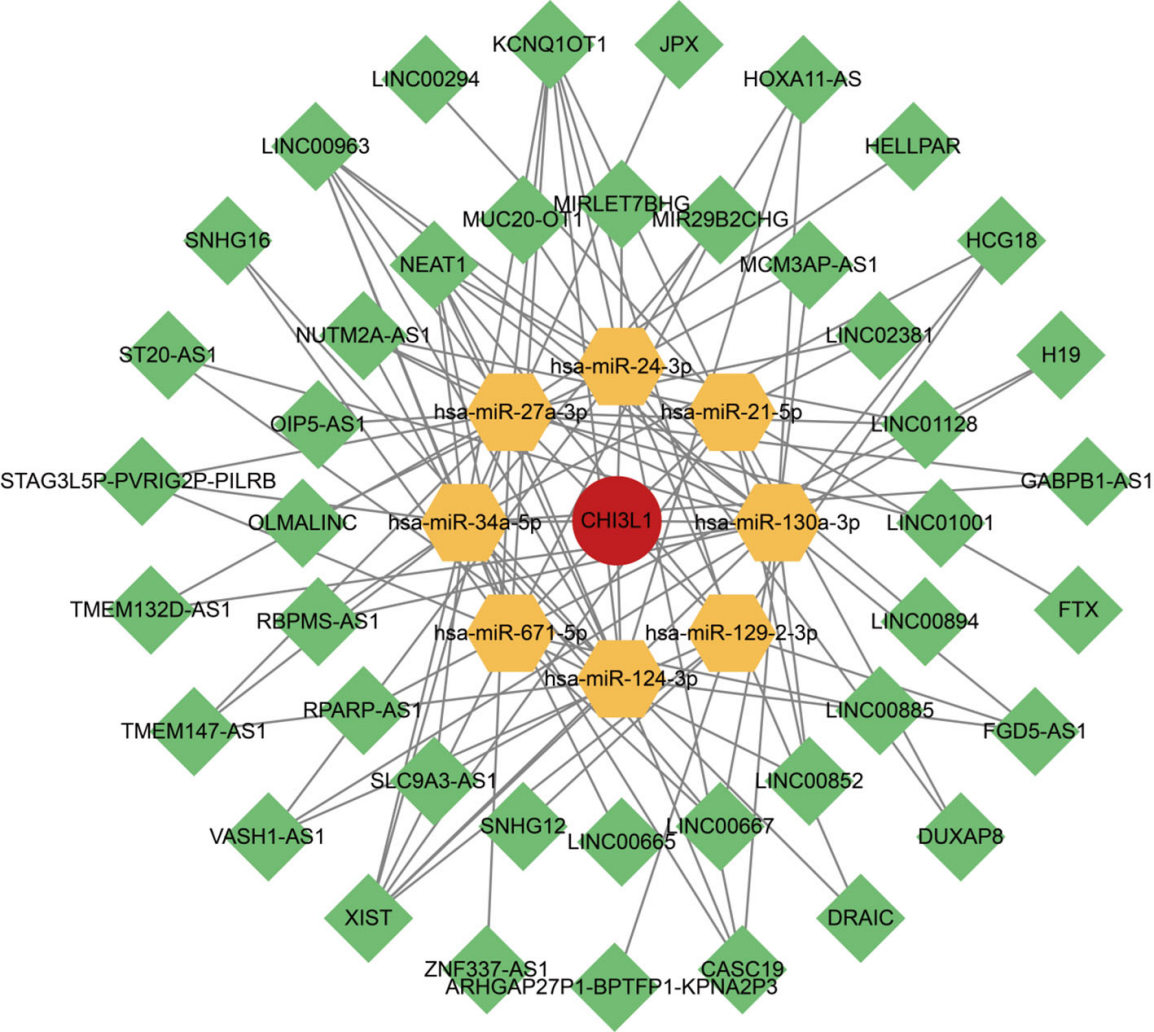
ceRNA regulatory network. The ceRNA regulatory network associated with CHI3L1. Red circular nodes represent CHI3L1, yellow hexagonal nodes represent miRNAs, and green diamond-shaped nodes represent lncRNAs.

The ceRNA network comprised one key mRNA, eight miRNAs, 43 lncRNAs, and 279 interaction pairs. Among them, lncRNAs such as DRAIC exhibited the highest degree values within the ceRNA network topology; miRNAs such as hsa-miR-21-5p and hsa-miR-24-3p showed the highest degree values among miRNAs; and CHI3L1 represented the mRNA with the highest degree value in the network (**Figure 7**).

### 3.8 Drug prediction

**Figure 8:**
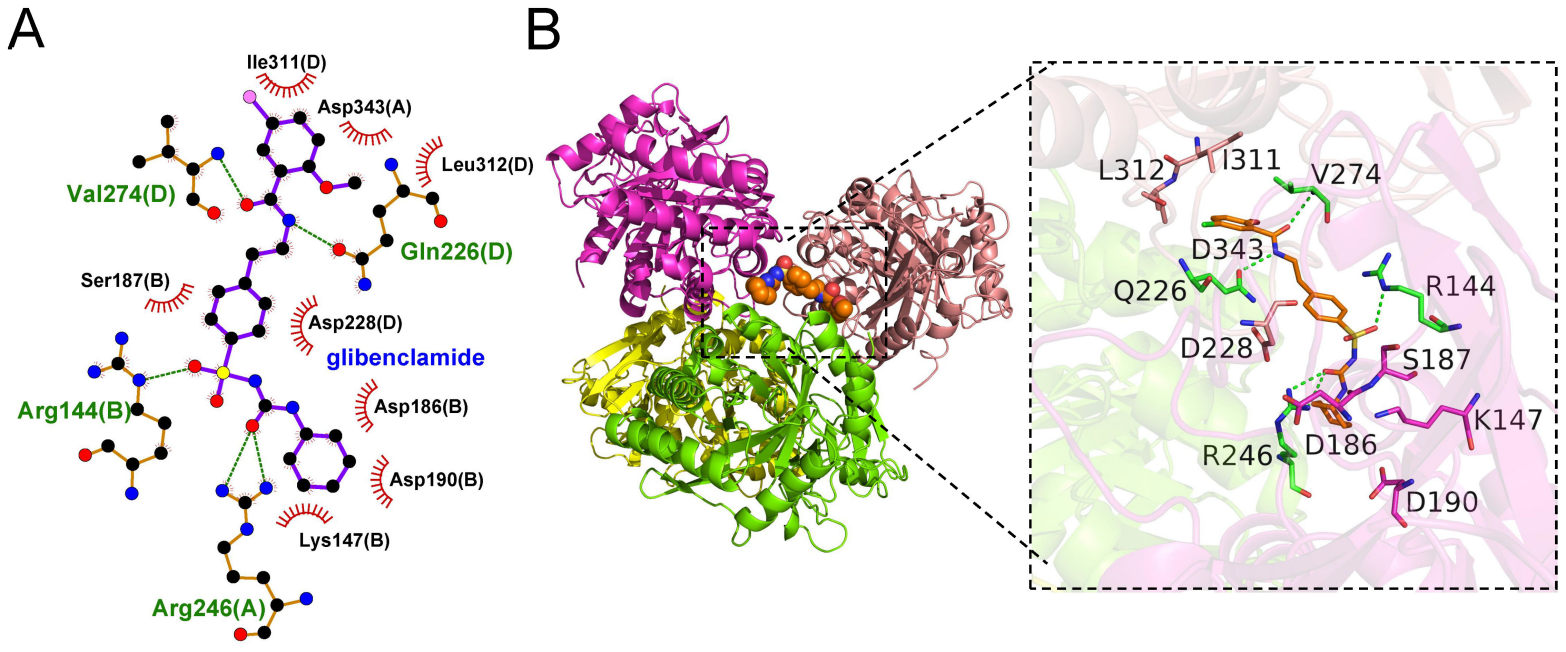
Molecular docking analysis of small-molecule drugs with the key target. **(A)** Two-dimensional visualization of the optimal ligand–receptor complex of CHI3L1, showing binding interactions between the small-molecule drug and the target protein. Green dashed lines indicate hydrogen bonds, and red gear-shaped symbols indicate hydrophobic interactions. **(B)** Three-dimensional visualization of the optimal ligand–receptor complex of CHI3L1.

By integrating predictions from the CMap and DSigDB databases, three candidate small-molecule drugs targeting CHI3L1 were identified: clindamycin (raw_cs = -0.4043, Binding free energies = -7.007 kcal/mol, primaquine (raw_cs = -0.2852, Binding free energies = -6.909 kcal/mol), and glibenclamide (raw_cs = -0.2852, Binding free energies = -9.386 kcal/mol), indicating favorable interactions between these compounds and the key target. Among them, glibenclamide exhibited the lowest binding energy (–9.386 kcal/mol) with CHI3L1 and formed hydrogen bonds and hydrophobic noncovalent interactions with residues IIe311, Asp343, and Leu312 in the active pocket region of CHI3L1 (**Figures 8A, B**).

### 3.9 Clinical validation of CHI3L1 in RA diagnosis and disease activity assessment

**Figure 9:**
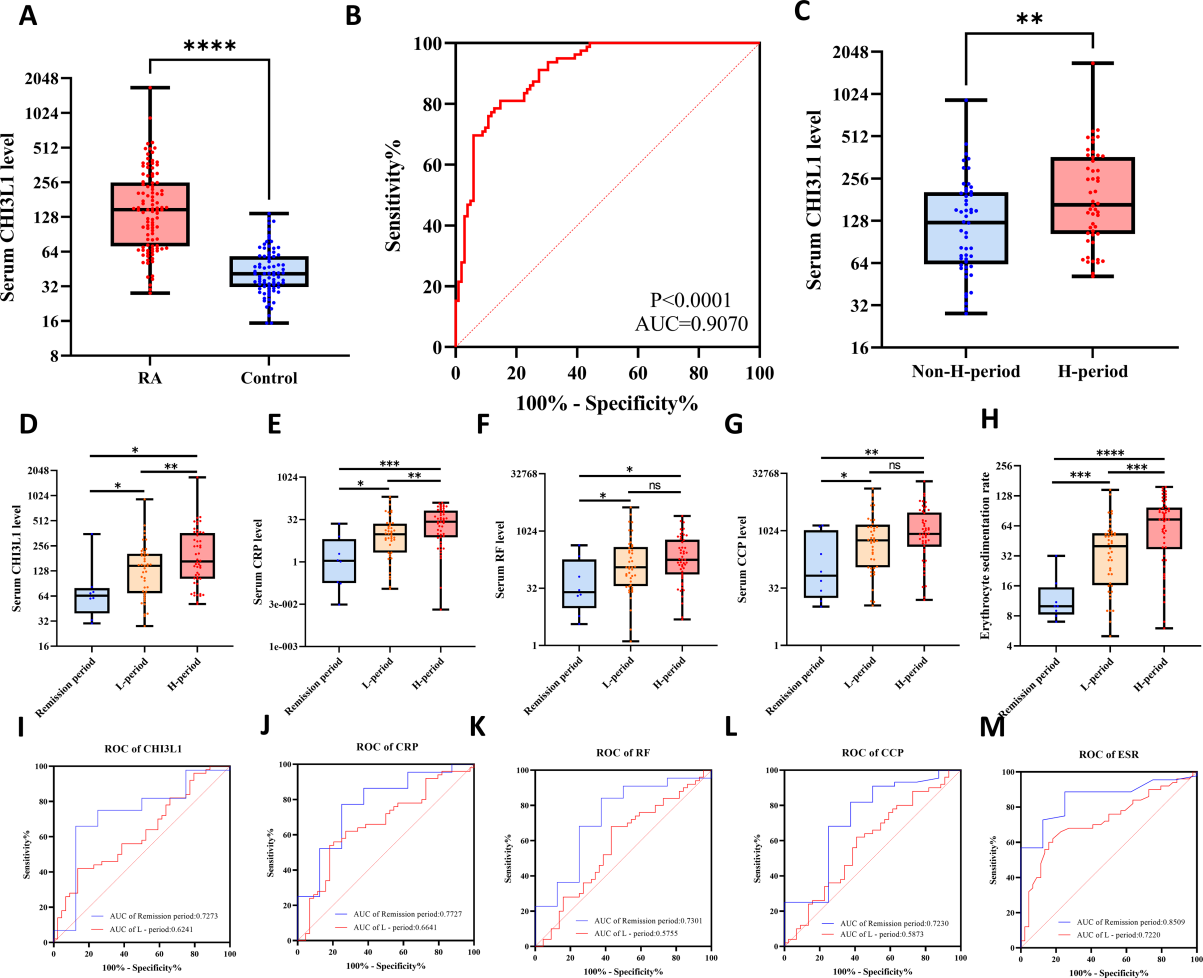
CHI3L1 as a biomarker for diagnosis and disease activity monitoring in RA. **(A)** CHI3L1 levels were significantly higher in RA patients (n=102) compared with healthy controls (n=79) (P<0.0001). **(B)** ROC analysis demonstrated robust diagnostic performance of CHI3L1, with an AUC of 0.907 (P<0.0001). **(C)** CHI3L1 levels were elevated in patients with high disease activity (DAS28-ESR >5.1) compared with those with low activity (P<0.01). **(D–H)** Comparison of CHI3L1, CRP, RF, CCP, and ESR among remission, low, and high activity groups. CHI3L1 and CRP increased progressively with disease activity. RF and CCP differentiated remission from active disease but not low from high activity. ESR showed the strongest correlation with disease activity, displaying a stepwise increase across groups. **(I–M)** ROC analyses of biomarkers in stratifying disease activity. ESR demonstrated the most consistent discriminative ability (AUC up to 0.8599), whereas CHI3L1 and CRP were more effective in distinguishing remission from low activity. RF and CCP showed limited power in differentiating low from high activity. *P<0.05, **P<0.01, ***P<0.001, ****<0.0001, ns: not significant (P>0.05).

Based on the preceding bioinformatics findings, we systematically validated CHI3L1 expression and its clinical significance in patient samples. CLIA results showed that serum CHI3L1 levels were significantly elevated in RA patients (n = 102) compared with healthy controls (n = 79) (P < 0.001) (**Figure 9A**). ROC curve analysis demonstrated excellent diagnostic performance of CHI3L1 for RA (AUC = 0.907, P < 0.0001) (**Figure 9B**). The analysis results of serum CHI3L1 and conventional biomarkers in patients with rheumatoid arthritis based on DAS28 disease activity are shown in **Supplementary Tables S3**, **S4** and **Supplementary Figure S2**. Further stratified analysis further revealed that CHI3L1 levels were markedly higher in patients with high disease activity (DAS28-ESR > 5.1) than in those with lower activity (DAS28-ESR ≤ 5.1) (P < 0.01) (**Figure 9C**), indicating its potential to reflect disease severity. Across different disease activity states, both CHI3L1 and CRP levels increased progressively with disease activity and peaked in the high-activity group (**Figures 9D, E**, **Supplementary Table S5**). These results suggest that CHI3L1 and CRP serve as effective biomarkers for dynamic monitoring of RA disease activity, with particular clinical value during high disease activity. By contrast, RF and CCP could distinguish remission from active disease but showed no significant differences between low- and high-activity groups (**Figures 9F, G**, **Supplementary Table S5**), limiting their utility in dynamic monitoring. ESR levels were strongly aligned with disease activity, exhibiting a stepwise increasing trend (**Figure 9H**, **Supplementary Table S5**). Further ROC analyses highlighted differences in the discriminative ability of each biomarker (**Figures 9I–9M**). ESR demonstrated the most stable performance in distinguishing remission from low activity (AUC = 0.8509) and low from high activity (AUC = 0.7220). CHI3L1 and CRP exhibited moderate to good diagnostic efficacy (AUC > 0.7) in differentiating remission from low activity, but their performance declined when distinguishing between low- and high-activity groups, suggesting greater utility in early disease monitoring. In comparison, RF and CCP retained limited diagnostic value, being effective only in distinguishing remission from active disease, but not across different levels of disease activity.

### 3.10 Correlation analysis of CHI3L1 with RA-specific antibodies and inflammatory markers

**Figure 10:**
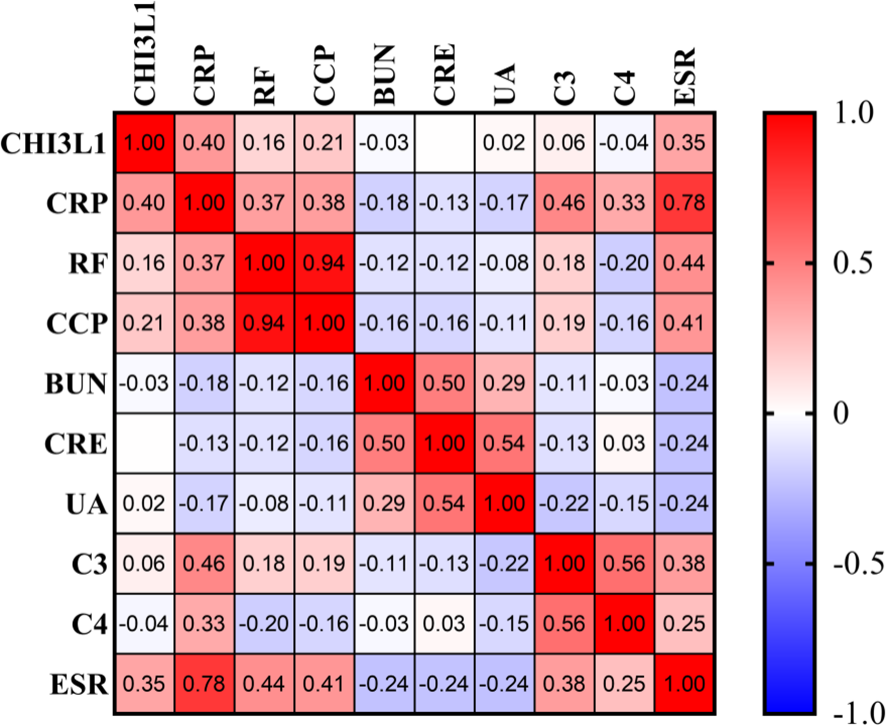
Correlation analysis of CHI3L1 with conventional serological inflammatory markers. Correlation heatmap showed the relationships among CHI3L1, RF, CCP, CRP, and ESR. CHI3L1 was positively correlated with CRP and ESR, and showed moderate correlations with RF and CCP. Correlation coefficients are represented by color intensity, with red indicating positive correlations and blue indicating negative correlations.

To further elucidate the potential role of CHI3L1 in inflammatory responses and immune processes in RA, we analyzed its correlations with inflammatory markers and RA-specific antibodies (**Figure 10**). CHI3L1 was positively correlated with CRP (r = 0.40, P < 0.001) and ESR (r = 0.35, P < 0.001), indicating its ability to effectively reflect the level of inflammation. In addition, CHI3L1 showed a moderate positive correlation with the RA-specific autoantibody CCP (r = 0.21, P < 0.05), further supporting its close association with RA-related immune responses. Moreover, RF and CCP were found to be highly correlated (r = 0.94, P < 0.001), reinforcing their consistency in RA diagnosis. CRP and ESR exhibited a moderate positive correlation (r = 0.78, P < 0.001), confirming their importance in assessing inflammatory activity.

## 4 Discussion

This study systematically elucidates the molecular regulatory role of CHI3L1 in RA and its close association with disease activity, while also confirming its diagnostic and prognostic potential through multilayered bioinformatics analyses and clinical validation. We demonstrated that CHI3L1 is significantly upregulated in both the serum and transcriptomic profiles of RA patients, with single-cell RNA sequencing revealing its predominant enrichment in synovial fibroblasts. Moreover, CHI3L1 expression correlated strongly with disease activity and conventional serological indicators. By integrating differential expression analysis, WGCNA, PPI mapping, immune infiltration profiling, and ceRNA network construction, we systematically uncovered the key molecular interactors, immunoregulatory mechanisms, and potential therapeutic targets associated with CHI3L1. Collectively, these findings highlight the central role of CHI3L1 in RA pathogenesis, suggesting that it not only promotes disease progression through modulation of inflammatory responses and synovial destruction but also holds promise as a molecular biomarker and therapeutic target with substantial translational potential.

### 4.1 Aberrant overexpression and cellular sources of CHI3L1 in RA

In this study, clinical samples combined with multi-omics analyses confirmed that CHI3L1 is markedly upregulated in RA. Serum CLIA assays demonstrated significantly elevated CHI3L1 levels in RA patients compared with healthy controls (P < 0.001), and ROC curve analysis indicated robust diagnostic performance (AUC = 0.907). These findings are consistent with previous reports in malignancies (28, 29), pulmonary fibrosis (30), and inflammatory bowel disease (31), suggesting that CHI3L1 may serve as a potential common molecular biomarker in inflammation-associated pathologies. Single-cell transcriptomic profiling further revealed that CHI3L1 is predominantly expressed in synovial fibroblasts. Given that synovial fibroblasts are recognized as key pathogenic cells in RA—capable of driving synovitis and tissue destruction through the secretion of chemokines, matrix-degrading enzymes, and inflammatory mediators (32, 33)—the enrichment of CHI3L1 in this cell population suggests a pivotal role in shaping the local microenvironment. However, our observation of fibroblast-dominant CHI3L1 expression appears to contrast with other studies reporting substantial upregulation in macrophages and neutrophils (34, 35), warranting critical examination. This discrepancy likely reflects context-dependent expression patterns influenced by disease stage and tissue microenvironment rather than contradictory mechanisms. This discrepancy likely reflects context-dependent expression patterns influenced by disease stage and tissue microenvironment rather than contradictory mechanisms. In acute inflammatory conditions, infiltrating macrophages and neutrophils responding to immediate tissue damage may serve as primary CHI3L1 sources (36). However, in established RA characterized by chronic synovitis and fibrotic remodeling, resident fibroblasts—which constitute approximately 50% of synovial lining cells (37)—likely become the major contributors to sustained CHI3L1 production. This transition aligns with RA’s progression from acute inflammation to chronic structural pathology. Supporting this concept, recent single-cell analysis of osteoarthritis identified distinct synovial fibroblast subsets with elevated CHI3L1 expression (38), demonstrating that fibroblast subpopulations can become primary CHI3L1 sources under chronic inflammatory conditions. Furthermore, CHI3L1’s ability to bind multiple ligands and activate diverse signaling pathways enables different cellular sources to utilize CHI3L1 for complementary functions: fibroblast-derived CHI3L1 may primarily regulate extracellular matrix remodeling and joint destruction, whereas macrophage-derived CHI3L1 may amplify inflammatory responses (39). Therefore, these findings collectively suggest that CHI3L1 operates through a multi-cellular network where different cell types predominate at distinct disease stages, with fibroblast-mediated production representing a hallmark of chronic inflammatory arthritides.

### 4.2 Molecular mechanisms and key genes: chemokine signaling, ECM remodeling, and metabolic regulation

Transcriptomic analysis identified 985 differentially expressed genes, of which 51 candidate genes overlapped with the 64 CHI3L1 co-expressed hub genes screened by WGCNA. Functional enrichment analysis revealed that these genes were mainly enriched in chemokine signaling pathways and mineral absorption processes. In RA, the chemokine pathway promotes immune cell recruitment and modulates the stability of the inflammatory microenvironment, thereby influencing disease progression (40, 41). Moreover, enrichment of the mineral absorption pathway suggests potential links between CHI3L1, iron metabolism, and oxidative stress, providing new insights into the persistence of inflammation and tissue damage in RA. PPI network analysis further highlighted TIMP1 and AQP9 as key genes associated with CHI3L1. TIMP1, a tissue inhibitor of metalloproteinases, plays a crucial role in regulating extracellular matrix (ECM) degradation and tissue remodeling, and is closely related to cell proliferation and the invasive phenotype of RA synovium (42). AQP9, a water channel protein, contributes to inflammatory cell migration and metabolic regulation, and is upregulated in a variety of inflammatory disorders (43, 44).

Our study demonstrated a significant positive correlation between CHI3L1 and both TIMP1 and AQP9, suggesting that CHI3L1 may exacerbate joint destruction and inflammation through a “fibroblast–ECM–immune cell” axis. Nevertheless, the current evidence is insufficient to establish a direct regulatory effect of CHI3L1 on TIMP1 and AQP9. Their association may instead be mediated by upstream signaling pathways such as NF-κB and IL-6/STAT3. Therefore, future studies employing genetic manipulation and functional assays are warranted to validate the causal relationships between CHI3L1 and these genes, and to delineate the precise role of CHI3L1 within the molecular network of RA.

### 4.3 Immunological mechanisms: interactions between CHI3L1 and immune infiltration

Immune cell infiltration within synovial tissue represents another hallmark of RA, where macrophages, T cells, and B cells exacerbate disease progression through cytokine secretion and autoantibody production (45). In our study, immune infiltration analysis revealed a significant increase in M0 macrophages and plasma cells, accompanied by a marked reduction in regulatory T cells (Tregs) and other immune subsets. This infiltration pattern is highly consistent with the persistent inflammation and impaired immune tolerance observed in RA (46, 47). Notably, CHI3L1 expression levels were strongly correlated with the degree of immune cell infiltration, suggesting that CHI3L1 may play a pivotal role in dynamically modulating the immune microenvironment. Previous studies have demonstrated that CHI3L1 can bind to receptors such as IL-13Rα2 and activate key signaling pathways including MAPK and PI3K–Akt, thereby promoting macrophage polarization and enhancing pro-inflammatory cytokine secretion (48, 49). In light of our findings, CHI3L1 appears not only to function as an inflammatory mediator secreted by synovial fibroblasts but also as a “bridging factor” that links structural cells with immune cells. Through this mechanism, CHI3L1 may facilitate immune cell recruitment and dysfunction, thereby amplifying inflammatory circuits and sustaining high-grade inflammation within lesions. Moreover, we observed a moderate positive correlation between CHI3L1 and the RA-specific autoantibody CCP, further supporting its association with RA-related immune responses. Given that plasma cells are the primary source of RA-specific autoantibodies, this finding suggests that CHI3L1 may indirectly drive autoimmune responses by promoting plasma cell differentiation or enhancing their functional activity. Importantly, this potential mechanism has not been systematically addressed in previous studies, providing novel insights and future directions for elucidating the immunological role of CHI3L1 in RA pathogenesis.

### 4.4 Clinical value: a novel biomarker for diagnosis and disease monitoring

Clinical validation demonstrated that CHI3L1 exhibits strong discriminatory power in the diagnosis of RA, with serum levels showing a stepwise increase in parallel with disease activity. Stratified analysis further revealed that CHI3L1 was particularly effective in distinguishing remission from low disease activity, providing important clinical reference for early identification and timely adjustment of therapeutic strategies. In contrast, although RF and CCP retain some diagnostic value in differentiating remission from active disease, their ability to discriminate across varying activity states remains limited. Compared with conventional inflammatory markers, CHI3L1—when used as a complement to CRP and ESR—shows promise for enhancing the sensitivity of disease monitoring. Moreover, incorporating CHI3L1 into a modified disease activity scoring system (e.g., DAS28-CHI3L1) could provide a more precise tool for disease management. Nonetheless, partial overlap between CHI3L1, CRP, and ESR under certain inflammatory conditions indicates that the independent predictive value of CHI3L1 requires further validation. Multivariable regression analyses and prospective cohort studies will be essential to clarify the additive benefit of CHI3L1 and to establish its clinical utility in RA management. Beyond conventional laboratory markers, CHI3L1 offers several distinct advantages. While RF and anti-CCP antibodies remain relatively stable throughout the disease course with limited responsiveness to activity changes, CHI3L1 levels track inflammatory fluctuations more dynamically. Additionally, whereas CRP and ESR lack disease specificity and can be influenced by various confounding factors, CHI3L1—being predominantly secreted by synovial fibroblasts—provides more RA-specific information reflecting local synovial pathology and tissue remodeling (11). Furthermore,CHI3L1’s mechanistic links to fibroblast activation, extracellular matrix remodeling, and immune infiltration enable it to integrate both structural and inflammatory aspects of RA pathophysiology (50, 51),potentially serving not only as a diagnostic tool but also as a prognostic indicator for joint destruction and treatment response. The combination of CHI3L1 with conventional markers may therefore establish a more comprehensive multi-dimensional assessment framework for precision RA management.

### 4.5 Therapeutic potential and drug prediction: from small molecules to RNA-based interventions

Molecular docking analysis revealed that among three candidate small molecules, glibenclamide exhibited the lowest binding energy with CHI3L1. As a classic sulfonylurea antidiabetic agent, glibenclamide has also been reported to possess anti-inflammatory and immunomodulatory properties (52–54). This finding provides new evidence supporting its potential repurposing in RA, suggesting that glibenclamide may exert additional therapeutic effects by targeting CHI3L1-related pathways. However, it is important to emphasize that drug prediction and molecular docking analyses only offer theoretical indications of potential interactions and cannot directly infer therapeutic efficacy. All candidate compounds require further validation in RA-relevant cellular models and animal studies, and the known risk of hypoglycemia and dose-dependent responses associated with glibenclamide must also be carefully considered. Therefore, the findings presented here should be regarded as hypothesis-generating rather than definitive evidence for clinical application.

At the transcriptional regulatory level, ceRNA network analysis identified multiple miRNAs and lncRNAs associated with CHI3L1, providing potential targets for RNA-based therapeutic strategies, including miRNA mimics, antisense oligonucleotides, and lncRNA modulation. These approaches could complement small-molecule interventions to establish a multidimensional therapeutic framework. However, RNA therapeutics still face critical challenges in delivery efficiency, off-target effects, and *in vivo* stability, which require further validation.

### 4.6 Limitations and future directions

This study elucidates the molecular regulatory role and clinical significance of CHI3L1 in RA; however, several limitations should be acknowledged. First, the absence of healthy synovial tissue controls in single-cell analysis represents a notable limitation. Due to ethical and technical constraints, healthy synovial biopsies are extremely difficult to obtain. To compensate, we employed cross-validation through bulk transcriptomic datasets with healthy controls and independent clinical validation. Second, the analyses primarily relied on transcriptomic and serological correlations, lacking direct validation from *in vitro* and *in vivo* functional experiments; therefore, causal relationships have not been fully established and is only indicative in nature. Third, although the clinical sample size was relatively adequate, the study was conducted at a single center, and the generalizability of the findings requires further confirmation in multicenter and multi-ethnic cohorts. Fourth, drug prediction and molecular docking analyses only indicated potential binding interactions, whereas the actual biological effects and therapeutic feasibility must be verified through cellular and animal experiments. To address these limitations, future studies should first establish healthy synovial single-cell reference atlases to enable rigorous comparative analyses of CHI3L1-related cellular interactions. Secondly, *in vitro* functional validation experiments will be conducted in CHI3L1 overexpressed synovial fibroblasts and peripheral blood mononuclear cell-derived macrophages to investigate whether glibenclamide inhibits the pathological processes related to rheumatoid arthritis (RA) by targeting CHI3L1. Subsequently, we should conduct comprehensive *in vivo* validation studies based on the RA animal model to clarify the pathogenic role of CHI3L1 in fibroblast dysfunction and immune cell recruitment, and to delineate its causal involvement in the amplification of inflammatory processes. Further investigation into the signaling interplay between CHI3L1 and key molecules such as TIMP1 and AQP9 will also be necessary to uncover more refined molecular regulatory networks. Building on these findings, targeted therapeutic strategies—such as neutralizing antibodies, small-molecule inhibitors, or RNA-based interventions—could be developed and evaluated in animal models for efficacy and safety, ultimately facilitating clinical translation. At the clinical level, incorporating CHI3L1 into modified disease activity scoring systems and validating its additive value in diagnosis, risk stratification, and therapeutic monitoring through multicenter prospective studies will be crucial for establishing its clinical utility.

## 5 Conclusion

This study revealed the aberrant upregulation and mechanistic role of CHI3L1 in RA. CHI3L1, predominantly secreted by synovial fibroblasts, interacts with TIMP1, AQP9, and immune cells to regulate chemokine signaling and extracellular matrix (ECM) remodeling, thereby promoting inflammation and disease progression. Clinical studies have shown that CHI3L1 has excellent performance in RA diagnosis and disease stratification, particularly reflecting disease activity in early monitoring. As a complement to conventional serological markers, CHI3L1 not only holds promise as a biomarker but also represents a potential therapeutic target. While this study provides important evidence for the mechanistic and clinical application of CHI3L1 in RA, its causal role and predictive value require further validation through multicenter prospective studies and functional experiments.

## Data Availability

The raw data supporting the conclusions of this article will be made available by the authors, without undue reservation.
