# Supplementary material for: Serum CHI3L1 levels correlate with disease activity in rheumatoid arthritis and reveal potential molecular mechanisms
Source: Front Immunol. 2025 Dec 18;16:1729989. doi: 10.3389/fimmu.2025.1729989 (PMC12756385; doi:10.3389/fimmu.2025.1729989)
Supplement: Supplementary file 1 [file DataSheet1.pdf]

Table S1. Baseline clinical and laboratory characteristics of patients with rheumatoid arthritis (n = 102)

**Clinical Characteristic, M (Q_1, Q_3)**	**Patients with rheumatoid arthritis (n = 102)**
Years	63.000 (57.000, 71.000)
ESR （mm/h）	50.000 (20.250, 83.250)
DAS28 score	5.054 (3.621, 6.251)
CHI3L1	147.655 (71.627, 253.532)
CRP (mg/L)	12.485 (3.095, 39.712)
RF (IU/mL)	140.550 (39.963, 389.222)
CCP (U/mL)	674.200 (166.950, 1663.625)
BUN (mmol/L)	5.320 (4.438, 6.328)
CRE (*u*mol/L)	54.000 (47.500, 67.750)
UA (*u*mol/L)	271.000 (214.750, 329.750)
C3 (g/L)	1.080 (0.903, 1.240)
C4 (g/L)	0.220 (0.190, 0.290)
IGG (g/L)	12.410 (10.440, 15.482)
IGA (g/L)	2.370 (1.600, 3.552)
IGM (g/L)	1.200 (0.905, 1.700)
WBC ($\times 10^9$/L)	5.960 (4.652, 7.433)
Absolute neutrophil count ($\times 10^9$/L)	4.155 (3.057, 5.232)
Absolute lymphocyte count ($\times 10^9$/L)	1.155 (0.903, 1.655)
Neutrophil-to-lymphocyte ratio (NLR)	3.234 (2.284, 4.582)
Gender, n (%)	
Male	30 (29.41)
Female	72 (70.59)
Activity, n (%)	
Remission	8 (7.84)
Mild activity	11 (10.78)
Moderate activity	33 (32.35)
High activity	50 (49.02)

Table S2. Differential Expression and FDR-Adjusted Statistical Results of CHI3L1, TIMP1, and AQP9 in Training and Validation Cohorts

gene	log2FC	P value	FDR（BH corrected ）
**Training cohort：**			
CHI3L1	0.328	0.00353	0.00529
TIMP1	0.358	0.000538	0.00323
AQP9	0.692	0.0046	0.00552
**Validation cohort：**			
CHI3L1	0.882	3.07E-08	9.21E-08
TIMP1	0.211	0.000444	0.000889
AQP9	1.39	1.23E-12	7.37E-12

Table S3. Baseline Characteristics of RA Patients by DAS28 Disease Activity

Clinical Characteristic	DAS28<5.1(n=52)	DAS28≥5.1(n=50)
Years		
60<	17(17%)	14(14%)
≥60	35(34%)	36(35%)
ESR（mm/h）		
50<	34(33%)	16(16%)
≥50	18(18%)	34(33%)
CHI3L1		
<148	30(29%)	22(22%)
≥148	22(22%)	28(27%)
CRP (mg/L)		
<12	32(31%)	17(17%)
≥12	20(20%)	33(32%)
CCP (U/mL)		
<674	32(31%)	19(19%)
≥674	20(20%)	31(30%)
RF (IU/mL)		
<141	31(30%)	20(20%)
≥141	21(21%)	30(29%)
Gender		
Male	16(16%)	14(14%)
Female	36(35%)	36(35%)

Table S4. Serum CHI3L1 and Conventional Biomarkers in RA Patients by DAS28 Activity

Clinical Characteristic	DAS28<5.1(n = 52)	DAS28≥5.1(n = 50)	Z	P
ESR mm/h	31.50 (13.75, 53.00)	74.00 (39.00, 96.50)	Z=-4.42	<.001
CHI3L1	124.37 (64.33, 201.75)	166.43 (103.49, 357.01)	Z=-2.71	0.007
CRP mg/L	7.50 (1.53, 18.19)	26.41 (7.68, 63.89)	Z=-3.44	<.001
RF IU/mL	101.59 (31.69, 329.22)	178.51 (78.81, 572.95)	Z=-1.84	0.066
CCP U/mL	536.20 (99.65, 1294.40)	848.00 (426.90, 2722.47)	Z=-2.05	0.041

Table S5. Comparison of Key Biomarkers Across Different Disease Activity Periods in Rheumatoid Arthritis

	Remission period	L-period	H-period
CHI3L1 M (Q_1, Q_3)	64.655 (52.972, 74.145)	147.325 (71.103, 205.585) ▲	166.430 (103.488, 357.012) ▲★
CRP mg/L M (Q_1, Q_3)	1.080 (0.185, 3.425)	9.580 (2.125, 20.823)▲	26.415 (7.680, 63.892)▲★
RF IU/mL M (Q_1, Q_3)	25.150 (16.657, 103.575)	113.115 (38.002, 376.070)▲	178.515 (78.807, 572.945)▲
CCP U/mL M (Q_1, Q_3)	67.850 (24.100, 516.350)	566.850 (114.950, 1349.6)▲	848.000 (426.900, 2722.475)▲
ESR mm M (Q_1, Q_3)	10.000 (8.750, 12.500)	40.000 (16.750, 54.000)▲	74.000 (39.000, 96.500)▲★

▲: There is a statistically significant difference compared to the Remission period (P<0.05)

★: There is a statistically significant difference compared to L-period (P<0.05)

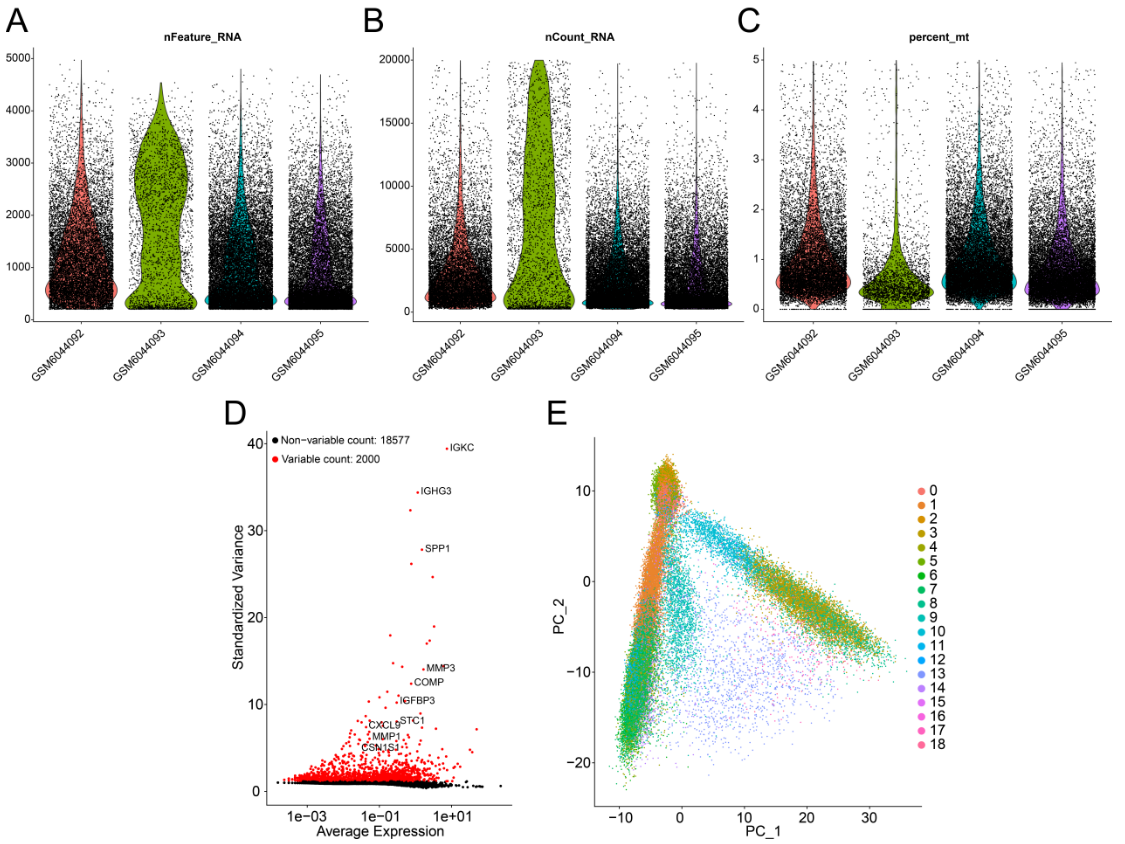

Figure S1. Characteristic distribution of single-cell RNA sequencing data after quality control and cell population clustering analysis.

(A) Violin plots showing the distribution of nFeature_RNA (number of detected genes) across four samples after quality control. (B) Violin plots showing the distribution of nCount_RNA (total RNA counts/UMI counts) across four samples after quality control. (C) Violin plots showing the distribution of mitochondrial gene expression percentage (percent_mt) across all samples after quality control. (D) Scatter plot showing highly variable gene selection results; highly variable genes are highlighted in red (n=2000) for downstream analysis. (E) Principal component analysis (PCA) plot showing cell clustering results based on PC1 and PC2.

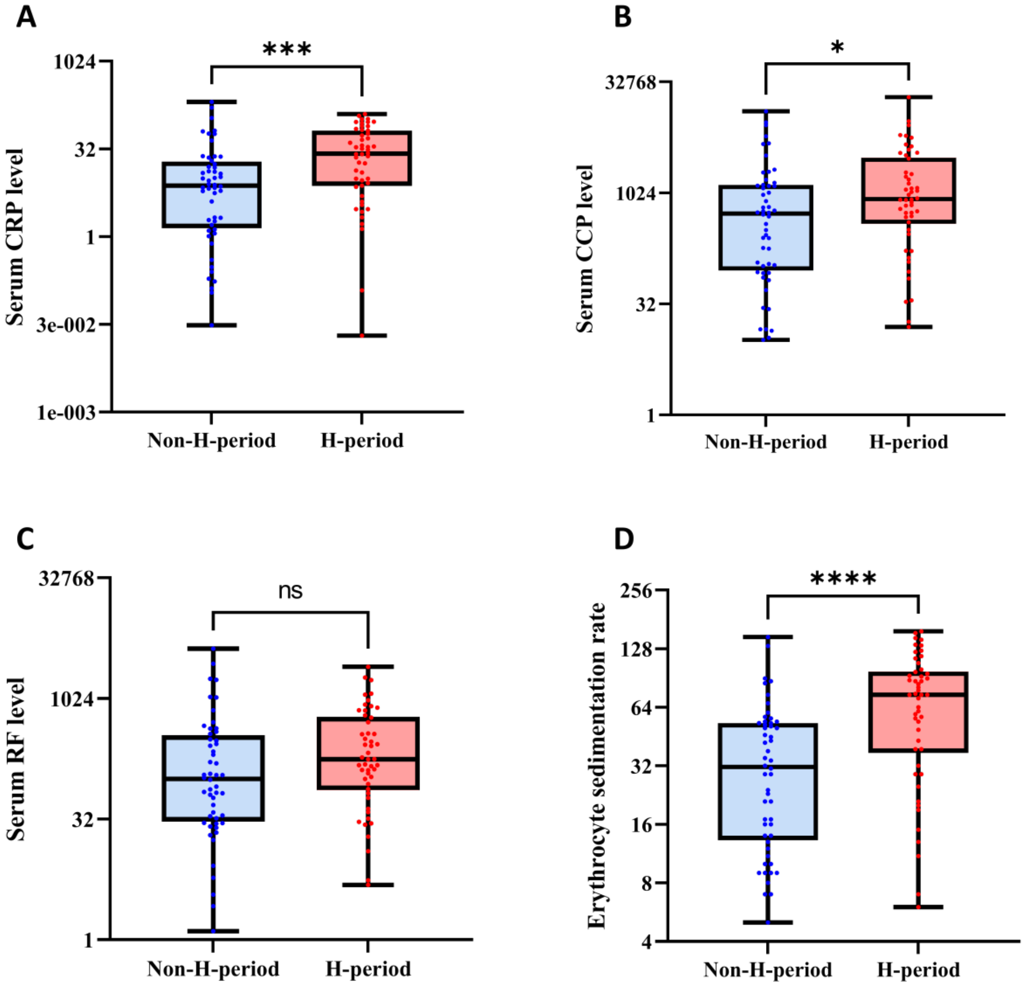

Figure S2. Distribution of (A) ESR, (B) CRP, (C) RF, and (D) anti-CCP antibody levels in RA patients stratified by disease activity (DAS28 <5.1 vs. ≥5.1).

Blue boxes represent DAS28<5.1 group and red boxes represent DAS28≥5.1 group. Median and interquartile ranges are shown. Statistical analysis was performed using Mann–Whitney U test. *P<0.05; ***P<0.001; ****P<0.0001; ns: P ≥ 0.05.
